# Bidirectional Cardio-Respiratory Interactions in Heart Failure

**DOI:** 10.3389/fphys.2018.00165

**Published:** 2018-03-06

**Authors:** Nikola N. Radovanović, Siniša U. Pavlović, Goran Milašinović, Bratislav Kirćanski, Mirjana M. Platiša

**Affiliations:** ^1^Pacemaker Center, Clinical Center of Serbia, Belgrade, Serbia; ^2^Faculty of Medicine, University of Belgrade, Belgrade, Serbia; ^3^Institute of Biophysics, Faculty of Medicine, University of Belgrade, Belgrade, Serbia

**Keywords:** cardio-respiratory coupling, heart failure, respiratory sinus arrhythmia, coherence, Granger causality, sample entropy

## Abstract

We investigated cardio-respiratory coupling in patients with heart failure by quantification of bidirectional interactions between cardiac (RR intervals) and respiratory signals with complementary measures of time series analysis. Heart failure patients were divided into three groups of twenty, age and gender matched, subjects: with sinus rhythm (HF-Sin), with sinus rhythm and ventricular extrasystoles (HF-VES), and with permanent atrial fibrillation (HF-AF). We included patients with indication for implantation of implantable cardioverter defibrillator or cardiac resynchronization therapy device. ECG and respiratory signals were simultaneously acquired during 20 min in supine position at spontaneous breathing frequency in 20 healthy control subjects and in patients before device implantation. We used coherence, Granger causality and cross-sample entropy analysis as complementary measures of bidirectional interactions between RR intervals and respiratory rhythm. In heart failure patients with arrhythmias (HF-VES and HF-AF) there is no coherence between signals (*p* < 0.01), while in HF-Sin it is reduced (*p* < 0.05), compared with control subjects. In all heart failure groups causality between signals is diminished, but with significantly stronger causality of RR signal in respiratory signal in HF-VES. Cross-sample entropy analysis revealed the strongest synchrony between respiratory and RR signal in HF-VES group. Beside respiratory sinus arrhythmia there is another type of cardio-respiratory interaction based on the synchrony between cardiac and respiratory rhythm. Both of them are altered in heart failure patients. Respiratory sinus arrhythmia is reduced in HF-Sin patients and vanished in heart failure patients with arrhythmias. Contrary, in HF-Sin and HF-VES groups, synchrony increased, probably as consequence of some dominant neural compensatory mechanisms. The coupling of cardiac and respiratory rhythm in heart failure patients varies depending on the presence of atrial/ventricular arrhythmias and it could be revealed by complementary methods of time series analysis.

## Introduction

The interaction of the cardiovascular and respiratory systems in healthy subjects has long been observed. It is thought that their relationship is determined by the autonomic nervous system and mainly reflected by respiratory sinus arrhythmia (RSA) (Schulz et al., 2013). Over the last few decades heart rate variability (HRV) analysis in time and frequency domain has been used as a noninvasive measure of RSA (Berntson et al., 1993; Task Force of the European society of cardiology the North American society of pacing electrophysiology, 1996). Recently, another type of cardio-respiratory coupling was recognized as an inverse phenomenon of RSA, i.e., cardio-respiratory phase synchronization (Schäfer et al., 1998; Lotrič and Stefanovska, 2000; Toledo et al., 2002; Bartsch et al., 2012). The duration of synchronization episodes varies according to physiological and pathological states and to the methods used for detection (Kuhnhold et al., 2017). Cardio-respiratory interaction has traditionally been perceived to be unidirectional via the influence of the respiratory rhythm on heart rate. However, several studies have revealed the existence of multi-directionality in the coupling of the cardiovascular and respiratory systems, and their dependencies on physiological and pathological conditions (Rosenblum et al., 2002; Mrowka et al., 2003; Porta et al., 2013; Platiša et al., 2016). Examination of the interactions of these two systems, quantification of their strength, and assessment of the pathways of coupling, are the subjects of increasing research focus. Studies including healthy subjects and patients have been conducted to better understand the regulatory and compensatory mechanisms, both those that are physiological and those that develop during disease progression (Schulz et al., 2013).

Autonomic nervous system imbalance is recognized in heart failure (HF), with increases in sympathetic activity and the withdrawal of vagal activity (Florea and Cohn, 2014; Hu et al., 2016). It is not entirely clear whether these autonomic nervous system changes are a cause or a consequence of HF; however, imbalances of the autonomic nervous system are expected to impact cardio-respiratory coupling, though this dynamic has yet to be investigated. This study aimed to examine the interactions of the cardiovascular and respiratory systems and their directionality in several groups of patients with HF.

## Materials and methods

### Subjects

We included patients with symptomatic HF and reduced left ventricular ejection fraction (LVEF < 35%) who had an indication for an implantable cardioverter defibrillator or cardiac resynchronization therapy device implantation. Patients were divided into three age- and gender- matched groups (Table 1) depending on whether they were in sinus rhythm (HF-Sin), sinus rhythm with ventricular extrasystoles (HF-VES), or permanent atrial fibrillation (HF-AF). In each group, we included 20 patients, five of whom were women. If the patient was in sinus rhythm and more than 20 premature ventricular ectopic beats were registered during signal recording, the patient was allocated to HF-VES group (Perez-Silva and Luis Merino, 2011). Of the 20 healthy control subjects, there were five women and 15 men (Table 1), and all were nonsmokers without any history of disease. They were instructed to avoid physical activity starting the day prior to measurement and to not eat or drink on the day of the examination.

**Table 1:** Cardiac and respiratory parameters.

	**Control**	**Heart Failure**		
		**HF-Sin**	**HF-VES**	**HF-AF**
	***N* = 20**	***N* = 20**	***N* = 20**	***N* = 20**
NYHA		2.20 ± 0.10	2.35 ± 0.11	2.50 ± 0.12
Age	44.0 ± 1.4^a^	64.5 ± 1.3	64.8 ± 1.9	66.2 ± 1.6
HR (bpm)	71.7 ± 2.3	63.5 ± 2.2^b^^,^^c^	75.2 ± 2.3	76.0 ± 3.4
BF (Hz)	0.225 ± 0.012^d^	0.255 ± 0.013	0.301 ± 0.019	0.282 ± 0.015
Coherence	0.773 ± 0.042^d^	0.598 ± 0.052^b^^,^^c^	0.353 ± 0.027	0.314 ± 0.031
SampEnRR	1.536 ± 0.072^a^	1.291 ± 0.094^b^	0.805 ± 0.082^e^	1.935 ± 0.042
SampEnResp	1.796 ± 0.054^g^	1.328 ± 0.086^h^	1.39 ± 0.10^f^	1.665 ± 0.074

a *p < 0.01 Control vs. all HF groups*.

b *p < 0.01 HF-Sin vs. HF-VES and HF-AF*.

c *p < 0.05 HF-Sin vs. Control*.

d *p < 0.01 Control vs. HF-VES and HF-AF*.

e *p < 0.01 HF-VES vs. HF-AF*.

f *p < 0.05. HF-VES vs. HF-AF*.

g *p < 0.01 Control vs. HF-Sin and HF-VES*.

h *p < 0.01 HF-Sin vs. HF-AF*.

*HR, heart rate; BF, breathing frequency. In each group there were 5 women. The one-way ANOVA with LSD post-hoc comparisons was used to compare all parameters between groups, except for coherence were we used Kruskall Wallis test with Mann Whitney U-test for post-hoc comparisons*.

### Data acquisition and processing

**Figure 1:**
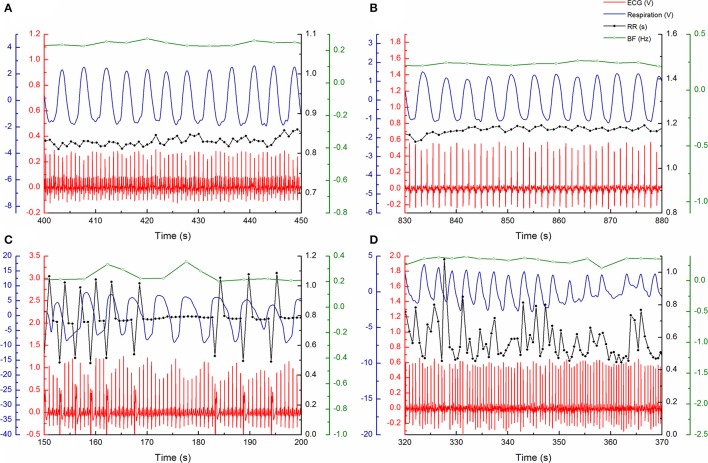
Examples of measured ECG (red) and respiratory (blue) signals and RR intervals (black) and respiratory rate (green) signals during arbitrary period of 50 s: **(A)** in 51 year old control subject, **(B)** 59 year old heart failure patient with sinus rhythm, **(C)** 62 year old heart failure patient with ventricular extrasystoles, and **(D)** 65 year old heart failure patient with atrial fibrillation.

Experiments were done in the morning, between 7:00 and 10:00 a.m., prior to device implantation in the HF patients, in a quiet setting at the Pacemaker Center of the Clinical Center of Serbia. Data were acquired from 20 min of signal measurements from relaxed subjects in the supine position and at spontaneous breathing frequency. We analyzed simultaneously recorded ECG and respiratory signals with a sample rate of 1 kHz and 16-bit resolution (BIOPAC System, Inc., Santa Barbara, CA, USA). The respiratory signal (Resp) was obtained from a transducer attached to the belt, which was used to measure abdominal expansion and contraction (Figure 1). Inter-beat (RR) intervals and inter-breath (BB) intervals were extracted from recorded signals using the Pick Peaks tool from OriginPro 8.6 (OriginLab Corporation, Northampton, MA, USA) (Figure 1). In patients with ventricular extrasystoles (VES), we used the coordinates of peak form VES as R peak coordinates for further analysis. Heart rate (HR) and breathing frequency (BF) were obtained as a reciprocal value of the mean RR and mean BB intervals. In addition, we resampled the respiratory signal according to the mean RR-values using linear interpolation between two neighbor samples. To obtain two series with the same number of points, we also performed equally equidistant resampling of the RR series using the mean RR-value for each subject. The resampling procedure was conducted as in the work of Kapidžić et al. (2014). Each equally resampled RR and Resp series was normalized by a standardized procedure; after subtracting the mean value, the demeaned series was divided by the standard deviation of the time series (Platiša et al., 2016).

### Cross power spectral analysis

Coherence is a measure of the relationship between two time series in the frequency domain that quantifies linear dependence between signals according to their similar frequency contents. Mathematically, it is defined by:

(1) $$\begin{array}{l} C_{x,y}\left(f\right)=\frac{{\left|P_{xy}\left(f\right)\right|}^{2}}{P_{xx}\left(f\right)P_{yy}\left(f\right)} \end{array}$$

where *P*_*xy*_ is the cross power spectral density of the two signals, *x* and *y*, while *P*_*xx*_ and *P*_*yy*_ are the power spectral densities of *x* and *y*, respectively, and *f* is the frequency in Herz (Hz). The estimation of the power spectral densities and cross-spectral density of the signals was carried out using the Welch method. The equally equidistant resampled RR intervals and Resp were broken down into epochs with a length of 256 points. A Hanning window was chosen, and an overlap of 50% with the next window was used. Coherence was estimated from the coordinates of the peak in the cross-power spectrum, which is at or near breathing frequency. It was computed as the inverse value of the mean breath-to-breath interval extracted from the respiratory signal for each subject. Analyses were done using OriginPro 8.6 (OriginLab Corporation, Northampton, MA, USA).

### Granger causality analysis

For a pair of two inter-related (stochastic) processes, the Granger causality approach is used to determine how much of the current value of one process can be explained by its past values and whether adding the lagged values of the second process can improve the explanation. Mathematically, a variable *x* causes a variable *y* if the information in the past of *x* helps predict the future of *y* with better accuracy than is possible when only the information in the past of *y* itself is considered. Two time series of the *x* and *y* dynamics can be presented as a linear bivariate linear regression model with prediction errors for each time series, ξ_1_ and ξ_2_ residuals.

(2) $$\begin{array}{l} x\left(t\right)=\overset{p}{\underset{j\;=\;1}{\sum }}A_{11,j}x\left(t-j\right)+\overset{p}{\underset{j\;=\;1}{\sum }}A_{12,j}y\left(t-j\right)+\xi_{1}\left(t\right)\\ y\left(t\right)=\overset{p}{\underset{j\;=\;1}{\sum }}A_{21,j}x\left(t-j\right)+\overset{p}{\underset{j\;=\;1}{\sum }}A_{22,j}y\left(t-j\right)+\xi_{2}\left(t\right) \end{array}$$

Logarithm of the ratio of the predicted errors variances [var (ξ)] for the restricted (R)—partial and unrestricted (U)—full model determined the magnitude of interaction.

(3) $$\begin{array}{l} F_{2\to 1}=\ln\;\frac{\mathrm{var}\left(\xi_{1R\left(12\right)}\right)}{\mathrm{var}\left(\xi_{1U}\right)} \end{array}$$

The model order (lag) *p*, representing the number of past observations to incorporate into the regression model, was chosen as the best according to the Akaike information criterion, in the range from 5 to 20 (Platiša et al., 2016). In this study the Granger causality approach in the time domain was applied on the equally equidistant resampled RR and Resp series to quantify the two-way causation of the RR and Resp signals. We used a MATLAB toolbox for Granger causal connectivity analysis with free downloadable tools provided by Seth (2010).

### Sample and cross-sample entropy

Sample entropy (SampEn) and cross-sample entropy (cross-SampEn) are non-linear measures of time series regularity derived from the probability of finding a similar pattern within signal/signals (Richman and Moorman, 2000). The signal with a small number of similar patterns is characterized by larger values of SampEn, which indicates its higher unpredictability (irregularity). Similarly, the coupling of two signals with a small number of similar patterns results in high cross-SampEn values, which indicates high asynchrony, i.e., a low association between analyzed systems. SampEn and cross-SampEn were calculated with fixed input variables *m* = 2 (window size) and *d* = 0.2 SD (tolerance, SD–standard deviation of time series) from the entire time series (approximately about 1,200 samples) using MATLAB codes downloaded from web site Physionet.org^1^.

### Statistical analysis

Statistical analyses were done using the software package SPSS (version 17.0, SPSS Inc., USA). Normal distributions of data were tested by the Shapiro-Wilk test in each group. Grainger causality and coherence values were not normally distributed. We used the Mann-Whitney *U*-test to identify differences between groups and the Wilcoxon test to compare bidirectional interaction. Other parameters showed normal distribution, and we used one-way ANOVA with the LSD *post-hoc* test to identify differences between groups. All data are reported as means ± standard error (SE). *P* < 0.05 were considered statistically significant.

## Results

Differences between groups in the basic parameters, heart rate and breathing frequency indicate alterations in the control of the cardiac and respiratory system induced by HF (Table 1). There was no statistically significant difference in NYHA class between the three groups of HF patients. HF-Sin patients had a lower average heart rate compared to the HF-VES and HF-AF patients (*p* < 0.01, both groups) and the control subjects (*p* < 0.05). Breathing frequency was higher in patients with arrhythmias, HF-VES and HF-AF (*p* < 0.01, both groups) compared with the control subjects. In HF patients, while there was significant difference in left ventricular ejection fraction (*p* < 0.01), no significant differences in heart and respiratory rate in regard to the NYHA class were shown (Table 2). The drugs used by the HF patients are indicated in Table 3.

**Table 2:** Cardiac and respiratory parameters of heart failure patients in regard to NYHA functional capacity.

**NYHA class**	**LVEF (%)**	**BF (Hz)**	**HR (bpm)**
II (*N* = 39)	27.3 ± 1.2^a^	0.268 ± 0.011	71.2 ± 2.2
III (*N* = 21)	22.0 ± 1.5	0.301 ± 0.017	72.4 ± 2.8

a *p < 0.01 NYHA II vs. NYHA III*.

*The t-test for independent samples was used. LVEF, left ventricular ejection fraction; BF, breathing frequency; HR, heart rate*.

**Table 3:** Therapy of heart failure patients shown by groups.

**Drug**		**HF-Sin (%)**	**HF-VES (%)**	**HF-AF (%)**
ACE inhibitor		19 (95)	19 (95)	18 (90)
ß–blocker	bisoprolol	7 (35)	10 (50)	8 (40)
	metoprolol	6 (30)	7 (35)	8 (40)
	carvedilol	6 (30)	3 (15)	3 (15)
	nebivolol	1 (5)	0 (0)	1 (5)
Aldosterone blocker		20 (100)	19 (95)	18 (90)
Loop diuretic		20 (100)	20 (100)	20 (100)
Amiodaron		6 (30)	12 (60)	7 (35)
Dilacor		2 (10)	1 (5)	8 (40)
Statin		12 (60)	13 (65)	12 (60)
Platelet inhibitors		8 (40)	9 (45)	5 (25)
Anticoagulants		6 (30)	7 (35)	19 (95)

*ACE, angiotensin-converting enzyme*.

Coherence, i.e., the linear measure of the relationship between cardiac and respiratory signals in the frequency domain–modulation of cardiac by respiratory rhythm, was the highest in healthy subjects and significantly reduced in all HF patients (*p* < 0.05). However, there was also a statistical difference between the HF-Sin and arrhythmia patients (*p* < 0.01, for both groups).

**Figure 2:**
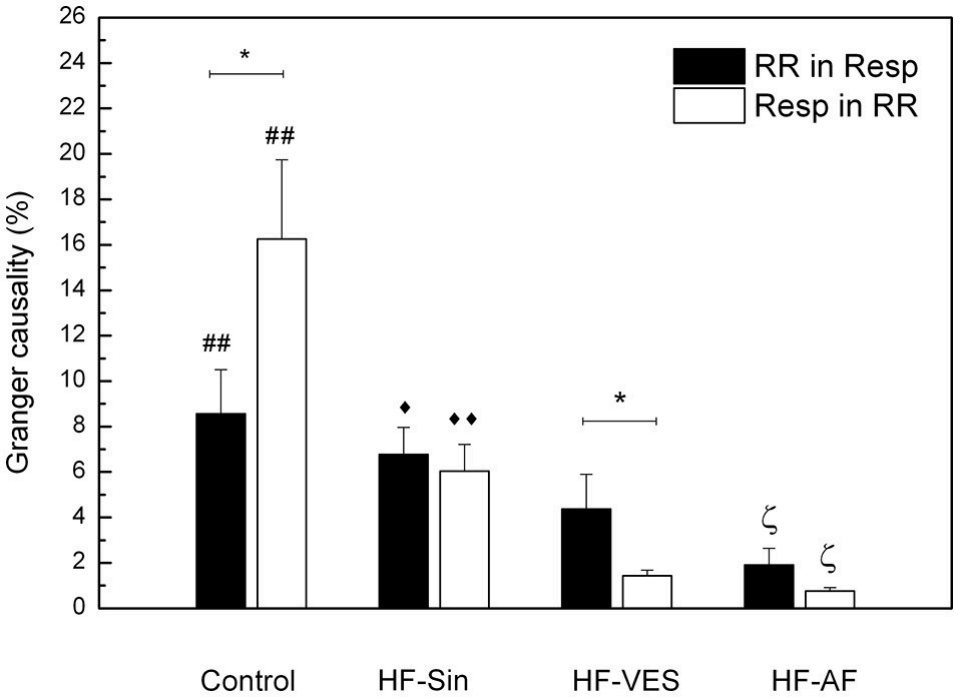
Bidirectional causality of cardiac rhythm in respiration (RR in Resp) and respiration in cardiac rhythm (Resp in RR). *p < 0.05 comparison in a group, ##*p* < 0.01, Control vs. HF-VES and HF-AF, ♦♦*p* < 0.01, ♦*p* < 0.05 HF-Sin vs. HF-VES ζ*p* < 0.05 HF-AF vs. HF-Sin and HF-VES.

In addition, we used Granger causality analysis to quantify the bidirectional interaction between the RR and respiratory signals. In healthy subjects, respiration was found to influence cardiac rhythm more significantly than the extent to which cardiac rhythm influenced respiration (*p* < 0.05), whereas we found that the influence of cardiac rhythm on respiratory rhythm was greater in HF-VES subjects (*p* < 0.05, Figure 2). In general, bidirectional influences were significantly reduced in HF-VES and HF-AF patients compared with control subjects (*p* < 0.01, for both groups). Among HF patients, the greatest reduction was found in the HF-AF group, with the least found in the HF-Sin patients (Figure 2).

As complementary measures, we used SampEn to estimate the irregularity of partial signals and cross-SampEn to determine any asynchrony between them. There was a significant difference in SampEnRR between all groups (*p* < 0.01; Table 1). The highest irregularity of cardiac rhythm was found in HF-AF patients and the lowest was found in HF-VES patients. Irregularity of the respiratory signal (SampEnResp) was the highest in healthy subjects and HF-AF patients, while the respiratory rhythm became more regular in the other two groups of HF patients (HF-Sin and HF-VES) (Table 1).

**Figure 3:**
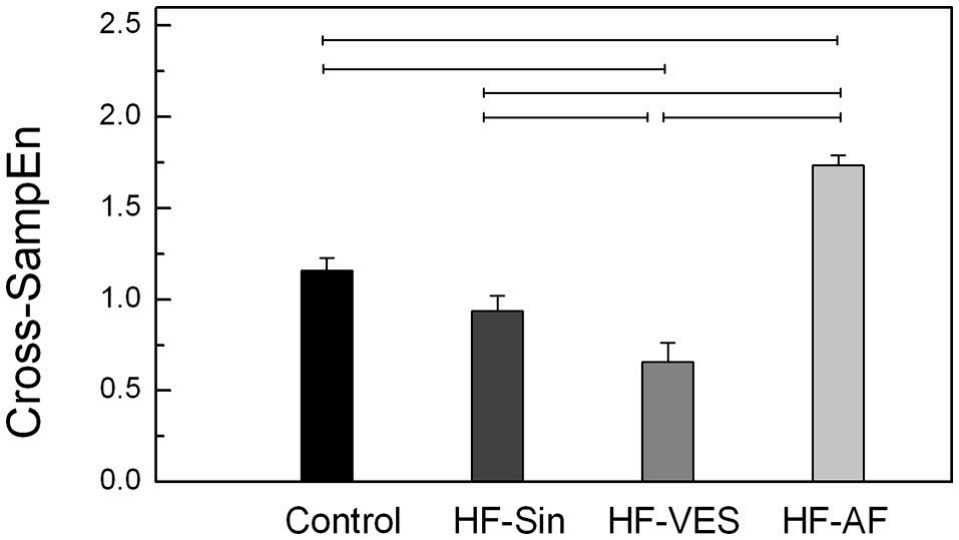
Cross-sample entropy (Cross-SampEn). Lines denoted significant difference, *p* < 0.05.

Synchrony was significantly different between healthy subjects and HF patients with arrhythmias, whereas there was no significant difference between the control subjects and HF-Sin patients (Figure 3). The strongest synchrony between respiratory and cardiac rhythms was found in HF-VES patients, while the weakest was found in HF-AF patients (Figure 3). Cross-SampEn was significantly lower in healthy subjects than in HF-AF patients but significantly higher than in HF-VES patients (*p* < 0.01).

## Discussion

To our knowledge, this is the first study aiming to determine the properties of cardio-respiratory coupling in HF using linear and non-linear methods of time series analysis. These complementary measures of the bidirectional interactions between RR intervals and respiratory rhythm yielded interesting results and a great deal of material for discussion.

Firstly, all HF patients in this study were receiving optimal therapy for HF according to ESC and ACCF/AHA/HRS guidelines. ß–blockers, angiotensin-converting enzyme (ACE) inhibitors, and aldosterone blockers are recommended for all patients with HF and reduced ejection fraction, and they all act primarily by modulating neurohumoral responses. In HF, there is extended and excess ß–adrenergic stimulation. In a normal heart, the ß1–receptors are predominantly situated on the cardiac sarcolemma and the ß2 in smooth muscle cells. However, the ß1–receptors in HF are down-regulated by the high circulating catecholamine levels. Since the cardiac ß2–receptors are not down-regulated, they are therefore increased in relative amounts, so that the ratio of ß1 and ß2–receptors shifts from 8:2 in a non-failing heart to 1:1 in HF (Barrese and Taglialatela, 2013). As a result of the increased activity of the adrenergic nervous system, stimulation of α1–receptors also occurs, which leads to peripheral vasoconstriction. In light of all of the above, it is not surprising that the results of the latest studies indicate that combined ß1–ß2–α blockers, such as carvedilol, are superior in the treatment of HF compared to the cardioselective (ß1–selectivity) agents, such as metoprolol or bisoprolol (Opie and Gersh, 2013). In our study, all HF patients were on ß-blocker therapy at the optimal or maximum tolerated dose. Many comorbidities including hypotension, bradycardia, worsening of already existing HF, asthma, and peripheral vascular disease necessitate the use of lower doses of ß-blockers, since cardioselectivity declines or is lost at high doses. In the present study, most patients used the highly ß1-selective agents, bisoprolol and metoprolol, while 12 patients were treated with carvedilol and 2 patients used nebivolol, a highly cardioselective drug with peripheral vasodilating properties mediated by nitric oxide. Carvedilol is the least potent antiarrhythmic drug among beta-blockers, and therefore it was used more frequently in the patients in the HF-Sin group than in the HF patients with arrhythmias. The role of the renin-angiotensin-aldosterone system (RAAS) in HF pathophysiology is crucial, and its overactivity is largely due to imbalances of the autonomic nervous system. As a result of reflex baroreflex activation that is caused by hypotension and exaggerated adrenergic stimulation, there is α-mediated peripheral vasoconstriction with an increase in peripheral vascular resistance and ß-mediated renin release with increased vasoconstrictive angiotensin II and aldosterone release (Opie and Gersh, 2013). ACE inhibitors have a permanent effect in decreasing angiotensin II and aldosterone, with declines in norepinephrine, epinephrine, vasopressin, and bradykinin that lead to vasodilatation, increased diuresis, and sodium excretion (Opie and Gersh, 2013). Although aldosterone blockers further reduce the effects of this final effector of RAAS and thereby decrease sodium and water retention, these drugs also have vasodilator properties and decrease the level of sympathetic tone. ACE inhibitors also improve parasympathetic activity, which is reduced in HF. In our study, only four HF patients did not use an ACE inhibitor, three due to hypotension and one because of terminal renal failure and hyperkalemia. That latter patient also did not use an aldosterone antagonist due to hyperkalemia, along with another two due to gynecomastia. All HF patients were treated with loop diuretics in order to provide symptomatic relief from fluid overload. Moreover, other antiarrhythmic drugs, in addition to ß-blockers, statins and antithrombotic agents were often used.

It is known that patients with HF have a faster resting ventricular rate compared to healthy controls (Hori and Okamoto, 2012). This is due to autonomic nervous system imbalance with increased sympathetic activity, which is the basic pathophysiological characteristic of this disease. However, our results prove that it is difficult to compare the heart rate of healthy controls and patients with HF, because all patients with HF and reduced ejection fraction are treated with ß-blockers, and many with additional antiarrhythmics, as is the case in our study, and their major effect is the control of heart rate (Li et al., 2015). Among patients with HF, HF-Sin patients have a significantly lower heart rate compared to HF patients with arrhythmias. In atrial fibrillation, since LV diastolic filling is reduced and thereby stroke volume as well, the increased heart rate, above 80 beats/min, represents a compensatory mechanism that enables the maintenance of adequate cardiac output (Cullington et al., 2014). Accordingly, and owing to the risk of developing of iatrogenic nocturnal pauses and pause-dependent ventricular tachycardia, there is a different, less strict therapeutic approach for patients with atrial fibrillation. Conversely, for patients with HF in sinus rhythm, lower heart rates (ideally below 70 beats/min) are associated with lower mortality and morbidity due to reduced myocardial oxygen consumption and improved myocardial efficiency (Cullington et al., 2014; Laskey et al., 2015). Heart rate in HF patients also depends on the stage of disease, and an elevated resting rate is associated with worse NYHA functional capacity. In our study, there was no statistically significant difference in heart rate between the patients in functional class NYHA II or NYHA III.

It is well-known that patients with HF have a higher respiratory rate compared to healthy controls, regardless of whether they are stable or decompensated (Goetze et al., 2015). Among HF patients, higher values of respiratory rate are correlated with worse NYHA functional capacity and lower left ventricular ejection fraction (Forleo et al., 2015). In our study, patients in functional class NYHA III had a significantly lower ejection fraction and higher respiratory rate, but the difference compared to patients in a lower NYHA class was not significant. While certain drugs and a high blood alcohol level can exert a depressiv effect on breathing with a subsequent decreased respiratory rate, this occurs primarily in the context of larger doses of sedatives, which was not the case in any of our patients. Furthermore, ß-blockers may trigger asthma exacerbations that are followed by a change in respiratory rate in susceptible patients; however, none of our HF patients had asthma.

Patients with HF often have enhanced carotid body chemoreflex sensitivity, and it is assumed that this increases sympathetic nerve activity, promotes respiratory-sympathetic coupling, and enhances respiratory rate and irregularity by increasing the frequency of oscillatory breathing, especially Cheyne-Stokes respiration (Marcus et al., 2014a,b). Among our HF patients, breathing frequency was higher in patients with arrhythmias. In previous studies, we have found an increased respiratory rate in patients with permanent atrial fibrillation compared to healthy subjects. We also concluded that in patients with imbalanced autonomic nervous system, a more regular heart rhythm is associated with a higher breathing frequency (Platiša et al., 2016). Indeed, in the present study, we found the lowest irregularity of cardiac rhythm, the lowest values of SampEnRR, and the highest respiratory rates in HF-VES patients.

The finding that coherence, defined as a linear calculation of cardiac modulation by respiratory rhythm, i.e., a measure of magnitude, was decreased in all HF patients was anticipated. There are two main reasons for this outcome. First, the pathophysiology of HF is characterized by imbalances of the autonomic nervous system leading to increased sympathetic activity and the withdrawal of vagal activity (Florea and Cohn, 2014). Thus, RSA, an index of cardiac vagal function, is predictably reduced in these patients (Yasuma and Hayano, 2004). Secondly, improves pulmonary gas exchange by matching perfusion to ventilation and stopping unneeded heartbeats during expiration (Yasuma and Hayano, 2004). Hence, reduction is a compensatory mechanism in patients with HF that leads to an increase in cardiac output. It should be noted that all drugs used in the treatment of HF, primarily ß–blockers, decrease the level of sympathetic tone and therefore enhance RSA, though it is never sufficient to regain the balance of the autonomic nervous system and normalize the RSA.

We found that heart rhythm was more regular in HF patients with sinus rhythm, with or without premature ventricular contractions, compared to healthy controls. This indicates that HRV, i.e., the beat-to-beat variation of the duration of the R-R interval, is reduced in HF patients (Billman, 2011). As HRV is strongly influenced by the autonomic nervous system and markedly complex dynamics of R-R intervals are considered a hallmark of health, this result is not surprising (Sassi et al., 2009; Billman, 2011). It is also not surprising that we found the highest irregularity of cardiac rhythm among HF-AF patients given to pathophysiological impact of atrial fibrillation. It has been shown that ß-blocker therapy can induce an increase in HRV parameters (Hoffmann et al., 1999; Aronson and Burger, 2001). In addition, some ACE inhibitors, such as captopril or lisinopril, improve HRV, and there is evidence that the same effect can be achieved by spironolactone, an aldosterone inhibitor (Kontopoulos et al., 1997; Yee et al., 2001). While it is clear that the effect of all these groups of drugs is a consequence of increased vagal tone, the persistently reduced HRV indicates that in HF, despite optimal therapy, no control of sympathetic activity can be established. The finding that the SampEnRR value was the lowest, indicating the highest cardiac rhythm regularity, in HF-VES patients requires further clarification. In healthy subjects, with preserved function of autonomic nervous system and baroreflex response, a premature ventricular ectopic beat provokes a biphasic reaction comprising an initial acceleration and late deceleration of the heart rate (Cygankiewicz, 2013). This phenomenon is known as heart rate turbulence, and it is responsible for heart rate irregularity in healthy subjects. In HF patients, there is an abnormal autonomic tone with a shift toward sympathetic overdrive, with decreased heart rate turbulence, which is a vagally-dependent effective measure of baroreflex sensitivity (Cygankiewicz, 2013). As a consequence, there is a lack of sudden acceleration and a blunted rate of subsequent deceleration, meaning that the ventricular ectopic beat does not contribute to heart rhythm irregularity. However, in future studies, a parallel investigation of both cardio-respiratory coupling and baroreflex sensitivity in several groups of HF patients is warranted.

The finding that respiratory rhythm was more regular in HF patients without atrial fibrillation than in healthy controls is the most challenging to discuss. In awake, healthy individuals at rest, there is diversity in the breathing pattern in terms of respiration rate, inspiratory and expiratory duration, and respiratory volumes (Benchetrit, 2000). Moreover, for the same person, breath-to-breath fluctuations in ventilatory variables exist. Studies have shown that this variability is not random and that each person has an individual resting breathing pattern, which can be regular or irregular, but that is reproducible in several conditions and over a long period of time (Benchetrit, 2000). On the other hand, breathing disorders are present in patients with HF, primarily Cheyne-Stokes respiration and obstructive sleep apnea. It is known that Cheyne-Stokes respiration appears significantly less often during wakefulness compared to sleep and that the onset of obstructive sleep apnea is not restricted to HF and can occur in the general population, mainly in obese subjects (Silva et al., 2008; Grimm et al., 2016). Hence, the finding that respiratory rhythms were more irregular in healthy controls than in HF patients is unexpected. However, when we take into account that the rate of breathing disorders is not so high in HF, that the onset of these disorders depends on many factors, including age, gender, obesity, NYHA class, and left ventricular ejection fraction, that the signal recording in our study lasted 20 min, and that a healthy person can have a regular or irregular breathing pattern, this result becomes less surprising. As previously noted, ß–blockers can affect the regularity of respiration, mainly by engendering a clinical worsening of asthma. Interestingly, there is some evidence that ß–blockers may be connected with decreases in the prevalence and severity of Cheyne-Stokes respiration (Silva et al., 2008).

An evaluation of the properties of cardio-respiratory coupling is not straightforward because the interaction of these two systems depends on many factors, including respiratory rate, age, and level of parasympathetic tone (Rosenblum et al., 2002). Moreover, it was previously found that there are other forms of cardio-respiratory coupling aside from the already described RSA, such as cardio-respiratory phase synchronization and time-delay stability, and that these coupling forms can coexist simultaneously (Bartsch et al., 2010). In previous research, as in the present study, it has been shown that there are significant bidirectional causal interactions between respiratory and RR signals in healthy subjects and that the influence of respiration on cardiac rhythm is significantly greater than that of cardiac rhythm on respiration (Platiša et al., 2016). The finding that bidirectional influences were significantly reduced in all HF patients due to the abnormal autonomic tone and reduced magnitude of RSA among these patients were expected. One notable finding was the greater influence of cardiac rhythm on the respiratory signal in all HF patients, especially the HF-VES group, than vice-versa. We believe that this result is a reflection of still insufficiently explored and understood compensatory mechanisms, which restore the regularity of heart rate and increase its impact on respiration in the context of the autonomic nervous system imbalance and impaired cardiac rhythm regulation by respiration. This hypothesis is supported by our results showing that HF-VES patients had the highest cardiac rhythm regularity and that the occurrence of premature ventricular ectopic beats in this group did not significantly reduce respiratory rhythm regularity. Another very interesting finding is that HF patients without atrial fibrillation and therefore with a significantly reduced RSA magnitude had increased bidirectional cardio-respiratory synchrony compared to healthy controls, with a strong influence of respiration on cardiac rhythm. In an examination of healthy subjects, Schäfer et al. found the same connection between these two signals and concluded that RSA and cardio-respiratory synchronization represent two different and competing aspects of cardio-respiratory interaction (Schäfer et al., 1998). While the occurrence of RSA is associated with baroreflex response, cardio-respiratory synchronization may be a result of a central coupling between cardiovascular and respiratory neuronal activities (Schäfer et al., 1998). Among the patients with HF and without atrial fibrillation, the strongest synchrony between respiratory and cardiac rhythms was observed in HF-VES patients, which once again confirms powerful effect of compensatory mechanisms.

Modulation and synchrony are different phenomena, and in terms of cardio-respiratory coupling, they can occur as a combination of both RSA and short-phase synchronization in healthy subjects or as two independent cardio-respiratory interactions in the context of longer phase synchronization, as seen in athletes (Schäfer et al., 1998; Lotrič and Stefanovska, 2000). Generally, in HF patients with arrhythmias, the ability to estimate RSA is limited. In HF-AF, it has background in the absence of RSA induced by dysfunction of the sinoatrial node; in HF-VES patients, reduced RSA is disturbed by the random appearance of ventricular extrasystoles. A greater number of ventricular ectopic beats will result in increased nonstationarity of the RR interval time series, and the condition of stationarity for the use of the linear method of analysis will not always be fully met. In the analyses of such nonstationary time series, the application of nonlinear methods yielded more accurate results. Hence, we can reliably confirm that the strongest synchrony between the respiratory and cardiac rhythms was found in HF-VES patients. On the other hand, similar results for the HF-Sin and HF-VES patients, obtained using linear time series analysis methods, indicate low levels of nonstationarity in the HF-VES data, suggesting reliable results of reduced coherence and causality between respiratory and cardiac rhythms.

In all four observed groups, there was the same gender distribution, and among the three groups of patients with HF, there were no statistically significant age differences. Gender differences in cardiac autonomic function are important until menopause, likely due to differences in hormonal status; in particular, estrogen levels have facilitating effect on cardiac vagal function (Voss et al., 2015). Moreover, aging is accompanied by significant modifications of the cardiovascular system and cardio-respiratory interactions (Ferrari, 2002). These changes occur due to increases in the sympathetic tone, decreased RSA, and the loss of baroreflex sensitivity that accompany aging, as well as increased vascular stiffness and peripheral resistance and a gradual uncoupling of respiratory activity and vagal outflow, which are also age-dependent processes (Porta et al., 2014). The discrete age differences that existed among our HF groups lose significance in light of the fact that age-induced differences of HRV, an index of balanced interplay of sympathetic and parasympathetic neural activity, begin disappearing after age 55 and are lost after age 65 (Voss et al., 2012). The average age of our patients in the HF groups was around 65 years. The main limitation of this study is that we did not age-match our HF patients and healthy control subjects. This is the expected limitation of this type of study, since it was not possible to select completely healthy subjects, especially those without any cardiovascular disease or risk factors for their development, among those who were closer in age to the HF patients. Furthermore, in our study protocol, patients did not drink water on the day of examination, which is not a common practice in autonomic nervous system function testing due to the potential development of artificially induced hypotension. However, since our patients proceeded to device implantation immediately after testing, they had to fast overnight. Lastly, it is important to note that we did not consider the possible presence of premature atrial complexes in our calculations, and their influence on cardio-respiratory interaction could be an interesting topic for a future study.

## Conclusions

In conclusion, our study has shown that the RSA magnitude and bidirectional causal interactions between the respiratory and RR signals are decreased in HF patients. We found that HF-VES patients have the highest regularity of cardiac rhythm, high respiratory rhythm regularity and the strongest synchrony between respiratory and cardiac rhythm. These results suggest that the role of compensatory mechanisms, especially in the presence of the autonomic nervous system imbalance, is significantly greater than previously thought.

## Ethics statement

All subjects gave written informed consent in accordance with the Declaration of Helsinki. The protocol was approved by the Ethics Committee of the Faculty of Medicine, the University of Belgrade (Ref. Numb. 29/III-5).

## Author contributions

NR, SP, GM, BK, and MP contributed to the study design, interpretation of data and drafting the manuscript. NR and MP collected and analyzed the data. All the authors read and approved the final manuscript.

### Conflict of interest statement

The authors declare that the research was conducted in the absence of any commercial or financial relationships that could be construed as a potential conflict of interest.

## Footnotes

^1^https://www.physionet.org/physiotools/sampen/matlab/1.1-1.
